# Active Poly(o-phenylenediamine)-Intercalated Layered δ-MnO_2_ Cathode for High-Performance Aqueous Zinc-Ion Batteries

**DOI:** 10.3390/polym17081003

**Published:** 2025-04-08

**Authors:** Ziqian Yuan, Bosi Yin, Wenhui Mi, Minghui Liu, Siwen Zhang

**Affiliations:** Institute of Clean Energy Chemistry, Key Laboratory for Green Synthesis and Preparative Chemistry of Advanced Materials, College of Chemistry, Liaoning University, Shenyang 110036, China; yuanziqian0128@163.com (Z.Y.); yinbosi@lnu.edu.cn (B.Y.); 18348634537@163.com (W.M.); liuminghui0922@163.com (M.L.)

**Keywords:** cathode materials, organic–inorganic composite, *δ*-MnO_2_, Zn-ion storage, hydrothermal synthesis

## Abstract

Aqueous zinc-ion batteries (ZIBs) represent an emerging energy storage solution that offers significant advantages in terms of safety, cost-effectiveness, and longevity in cycling. Among the various materials available, manganese-based oxides stand out as the most promising options for cathodes due to their impressive theoretical specific capacity, suitable operating voltage, and abundant natural availability. In published reports, pre-embedding is frequently used to modify the layered cathode; however, non-electrochemically active molecular embedding often results in a decrease in battery capacity. In this paper, a hydrothermal method is employed to intercalate poly(o-phenylenediamine) (PoPD) into δ-MnO_2_ (MO) to produce PoPD-MO cathode materials. Here, PoPD serves a dual role in the cathode: (1) PoPD is inserted into the interlayer of MO, providing support within the intercalation layer, enhancing material stability, increasing ionic storage sites, and creating space for more Zn^2+^ to be embedded, and (2) inserting PoPD into the interlayer structure of MO effectively expands the space between layers, thus allowing for greater ion storage, which in turn enhances the rate and efficiency of electrochemical reactions. Consequently, PoPD-MO shows remarkable cycling durability and adaptability in ZIBs, achieving a specific capacity of 359 mAh g^−1^ at a current density of 0.1 A g^−1^, and even under the strain of a high current density of 3 A g^−1^, it maintains a respectable capacity of 107 mAh g^−1^. Based on this, PoPD-MO may emerge as a new cathode material with promising applications in the future.

## 1. Introduction

To reduce the dependence on primary energy sources and relieve the pressure of extracting primary resources, the development of secondary energy sources has become our most important task now [1,2,3]. Among these, lithium-ion batteries (LIBs) garnered considerable attention, primarily thanks to their exceptional theoretical capacity and extended durability [4]. However, the earth’s limited supply of lithium, coupled with safety concerns regarding the electrolyte, has thrown a wrench in the gears of their advancement [5,6]. Therefore, people have to look for alternative sources of energy. Aqueous zinc-ion batteries (ZIBs) have become a prominent choice among many alternative resources due to their low cost and safe electrolyte [7,8]. However, the ion de-intercalation electrochemical reaction that occurs on the cathode material of ZIBs is the key to determining the battery performance [9,10,11]. Currently, the main cathode materials for ZIBs include vanadium-based materials [12,13,14,15], manganese-based materials [16,17], and Prussian blue analogs [18]. Manganese-based compounds have emerged as the leading contenders in cathode material research, primarily owing to their widespread availability, low pollution, and versatile oxidation states [19]. Unlike other manganese-based cathode options, manganese dioxide stands out for its diverse crystalline structures [20]. These advantages make it a popular choice for cathode material in ZIBs, owing to its simple preparation process and impressive theoretical specific capacity. Of all the MnO_2_ crystal structures, δ-MnO_2_ (MO) features a wider layer spacing, which is theoretically more suitable for the insertion of cations and therefore more advantageous [21]. Unfortunately, MO cathodes have the disadvantages of easy structural collapse and poor stability of the charging and discharging process during long-term cycling, resulting in a limited cycling lifetime [22]. To address these issues, researchers have attempted various methods to improve the electrochemical performance of MO [23,24]. Ultimately, it has been found that pre-intercalation is particularly effective for layered materials [25]. In recent years, cation intercalation and polymer molecule intercalation have been commonly employed to enhance the performance of MO. For example, the introduction of Na^+^ into the MO structure can offer a promising strategy to optimize ion transport and stabilize the framework [26]. Chomkhuntod et al. used a highly charged pre-intercalator (Al^3+^) to reduce interaction between Zn^2+^ and cathode material [27]. This approach facilitated a highly reversible intercalation of Zn^2+^, culminating in impressive capacity and cycling stability. However, despite the fact that cation doping can offer some structural stabilization for manganese-based materials, it can also lead to lattice distortion stress due to issues related to ionic radius and charge [28]. Therefore, it remains essential to explore alternative substances to further enhance MO cathode efficiency.

Recently, Huang and Wang et al. used polyaniline (PANI) to intercalate into MnO_2_ for ZIBs, which enlarged the intralayer space and enhanced the stability of the material [29]. However, PANI often needs to be in a highly acidic environment to exhibit high activity, yet high concentrations of H^+^ can damage zinc metal. Despite subsequent further modifications, such as the introduction of dibenzoic acid into PANI [30] or the composite of PANI with graphite material [31], all of them exhibited low capacity (180 mAh g^−1^) with cycling instability (70% capacity retention). Zhang et al. presented the first poly(o-phenylenediamine) (PoPD) for ZIBs. Unlike other polymer types, PoPD has a phenazine-like trapezoidal structure, and the phenazine ring of PoPD can be coordinated with divalent zinc ions via conjugated C=N bonds during charging–discharging [32], and its excellent conjugated structure provides a stable voltage platform for ZIBs. However, in weakly acidic solutions, the organic PoPD is easily dissolved. Zhang et al. synthesized N-doped vanadium oxide cathode materials using an organic–inorganic pre-intercalation strategy, where N doping and oxygen vacancies simultaneously increased the electronic conductivity and accelerated the diffusion kinetics of zinc ions [33]. Therefore, we consider the composite of PoPD with inorganic materials, which may become an efficient cathode for ZIBs.

In this study, an organic–inorganic composite cathode material (PoPD-MO) was obtained by intercalating electrochemically active poly(o-phenylenediamine) (PoPD) into manganese dioxide. Various characterization tests and analyses revealed that the insertion of PoPD provides a supporting role within the interlayer, expanding the interlayer spacing while enhancing structural stability and reducing the resistance during Zn^2+^ insertion and extraction. Additionally, the unique phenazine rings in PoPD can coordinate with Zn^2+^ through conjugated C=N bonds, improving battery capacity and maintaining the stability of the voltage plateau. Leveraging these advantages, electrochemical tests of the PoPD-MO cathode in zinc-ion batteries demonstrated a remarkable specific discharge capacity of 107 mAh g^−1^ at 3 A g^−1^, with a capacity retention of 91.3% over 2100 cycles. These findings open new avenues for developing innovative methods for organic–inorganic cathode materials in advanced energy storage systems.

## 2. Results and Discussion

**Figure 1:**
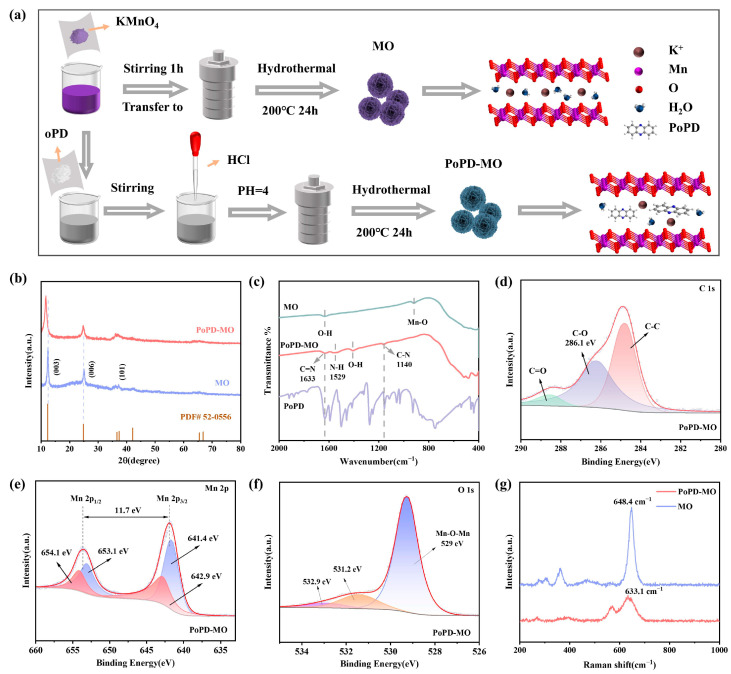
(**a**) Flowchart for the synthesis of PoPD-MO and MO. (**b**) XRD comparison images of PoPD-MO and MO. (**c**) FTIR spectra of PoPD-MO and MO. (**d**) C 1s, (**e**) Mn 2p, and (**f**) O 1s in the XPS spectra of PoPD-MO. (**g**) Raman spectrum of PoPD-MO and MO.

Figure 1a shows the synthesis process of the δ-MnO_2_ cathode and PoPD intercalation of the δ-MnO_2_ (PoPD-MO) cathode material, in which o-phenylenediamine monomer is polymerized in manganese dioxide crystals, leading to a decrease in the amount of K^+^ and water of crystallization, and PoPD is intercalated into the intercalation of MO crystals. X-ray diffraction tests (XRD) were performed on PoPD-MO and MO, as depicted in Figure 1b. The XRD diffraction peaks of MO and PoPD-MO are basically consistent with those of birnessite-type δ-MnO_2_ (JCPDS No. 52-0556) [34], and no impurity peaks were observed, indicating that MO and PoPD-MO with monoclinic crystal structure were successfully synthesized. Compared with MO, the characteristic peak corresponding to the (003) crystal plane of PoPD-MO shifted leftward and the diffraction angle changed from 12.34° to 11.61°. The peak shape also changed accordingly, which was manifested as a decrease in peak intensity and an increase in peak width, indicating that the addition of PoPD reduced the crystallinity of the material [35]. Calculated according to Bragg’s law (2dsin*θ* = nλ) [36], the spacing of the crystalline plane of (003) widened from 7.1 Å to 7.6 Å, which suggests that the insertion of PoPD did not change the crystalline structure of the sample but rather increased the layer spacing. From this, it can be inferred that the monomer o-phenylenediamine is likely to polymerize between the MO layers, thus achieving the expansion of the layer spacing without destroying the original crystal structure. To further analyze the intercalation mode of the polymer PoPD into MO and the functional groups present in the two samples, Figure 1c shows our Fourier transform infrared spectroscopy (FTIR) tests of MO and PoPD-MO. Compared with the FTIR spectra of MO, the PoPD-MO material displays distinct peaks at wavelengths of 1633, 1529, and 1140 cm^−1^, which exhibit significant differences from MO. Notably, the peaks at 1633 and 1140 cm^−1^ align with the C=N and C-N bonds stretching vibrations in the PoPD-MO structure [32]. Additionally, the peak at 1529 cm^−1^ arises from the characteristic vibrational motion of the aromatic benzene ring [37]. Hence, successful preparation of PoPD-MO can be demonstrated. In addition, the electronic structures and elemental compositions of the two cathodes were also tested by X-ray photoelectron spectroscopy (XPS). Figure 1d shows the C 1s XPS spectra of PoPD-MO, where peaks at 284.8, 286.1, and 288.6 eV can be attributed to C-C, C-O, and C=O bonds [38]. Figure S1a (Supplementary Materials) shows the C 1s spectra of MO. Compared with MO, PoPD-MO has a stronger characteristic peak at 286.1 eV. This is related to the C-O and C-N bonds formed after PoPD intercalation [39]. High-resolution XPS spectra of PoPD-MO and MO (Figure 1e and Figure S1b, Supplementary Materials) show that the two main peaks of Mn 2p are positioned at 641.9 and 653.6 eV, representing the Mn 2p_3/2_ and Mn 2p_2/1_ energy levels, and spin–orbit splitting is 11.7 eV [40]. In addition, the two main peaks of PoPD-MO can be de-convoluted to 641.4, 653.1 eV, and 642.9, 654.1 eV attributed to Mn^3+^ and Mn^4+^ [41]. Compared with the original MO, the proportion of Mn^3+^ was significantly increased in PoPD-MO, suggesting that the embedding of PoPD could result in oxygen vacancies [40]. In addition, Figure 1f and Figure S1c (Supplementary Materials) display O 1s XPS spectra for PoPD-MO and MO, respectively. Among them, the peaks at 529, 531.2, and 532.9 eV correlate with Mn-O-Mn bonds, oxygen vacancies, and surface-adsorbed water [42]. The oxygen vacancy peak (531.2 eV) of PoPD-MO was significantly enhanced compared to the original MO, suggesting that the insertion of PoPD led to an increase in the concentration of oxygen-deficient sites [16]. Figure 1g illustrates the Raman spectra for the two cathodes, where the peaks in the 625–650 cm^−1^ range are generated by the vibrations of the v_2_ (Mn-O) bonds in the MnO_6_ group. The peak observed in the 560–580 cm^−1^ range comes from the telescopic vibrations of v_3_(Mn-O) within the [MnO_6_] plane [43]. Compared with the peak of MO at 648.4 cm^−1^, the intensity of the Raman peak of PoPD-MO at 633.1 cm^−1^ is substantially reduced, while the telescopic vibrational peak of v_3_(Mn-O) bond is more obvious, suggesting that the gravitational force between manganese and oxygen is enhanced, which may be caused by the creation of oxygen vacancies [44]. In addition, the Raman peak of PoPD-MO at 633.1 cm^−1^ is significantly blue-shifted, which is attributed to the increased layer spacing after PoPD intercalation, which facilitates the rapid migration of Zn^2+^, and ultimately realizes the capacity enhancement [45].

**Figure 2:**
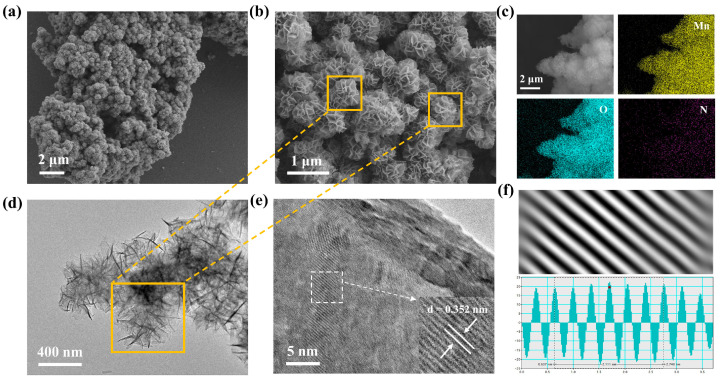
(**a**,**b**) SEM images of PoPD-MO. (**c**) Elemental mapping images of PoPD-MO. (**d**) TEM image of PoPD-MO. (**e**,**f**) HR-TEM images of PoPD-MO.

To observe the morphology of MO and PoPD-MO and to confirm that PoPD is embedded in the interlayers of MO, the morphological features and microstructures of two cathodes were characterized by scanning electron microscopy (SEM) and transmission electron microscopy (TEM). Figure 2a,b and Figure S2a,b (Supplementary Materials) depict the scanning electron microscope (SEM) images of PoPD-MO and MO at different magnifications, where both PoPD-MO and MO show 3D flower-like porous nanostructures composed of nanosheets. Compared with MO, the particle size of PoPD-MO is significantly reduced, which is mainly because the introduction of PoPD promotes the creation of oxygen vacancies, and these defects modify the crystals’ inherent stress state, resulting in a shift toward a denser crystal structure, and ultimately leads to a decrease in the radius [46]. In addition, the spheres in PoPD-MO were dispersed more uniformly without obvious agglomeration, indicating that the insertion of PoPD is favorable to the maintenance of the nanoflower structure, making it more compatible with the embedding and detachment of Zn^2+^. Furthermore, energy dispersive spectroscopy (EDS) provided clear evidence of Mn and O elements within the MO framework (Figure S3, Supplementary Materials). Figure 2c and Figure S4 (Supplementary Materials) also demonstrated the homogeneous dispersion of Mn, O, and N across the nanostructures of PoPD-MO, solidifying the presence of PoPD in the composite. Additionally, the transmission electron microscopy (TEM) images of the synthesized samples are presented in Figure 2d and Figure S5a (Supplementary Materials), in which PoPD-MO is a nanoflower-like structure assembled from nanosheets with diameters of about 400 nm, aligning well with the findings from the SEM tests. High-resolution transmission electron microscopy images (HR-TEM) (Figure 2e,f and Figure S5b, Supplementary Materials) show clear lattice striations of PoPD-MO and MO. We can observe that the interlayer spacing of MO is 0.289 nm, which corresponds to the (101) crystallographic plane. The introduction of PoPD increases the interlayer spacing to 0.352 nm. This expansion may stem from the introduction of PoPD, which acts as an interlayer intercalation of the sample, which enhances the stability of the structure, facilitates the embedding and dislodging of Zn^2+^, and increases the ion diffusion rate. Therefore, the above microscopic morphology analysis of the prepared material proves that we successfully synthesized PoPD-MO with a nanoflower-like structure.

**Figure 3:**
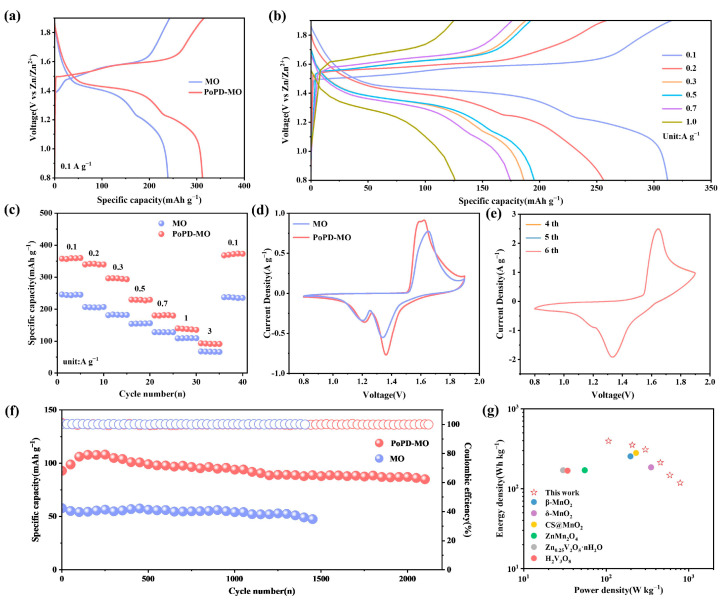
(**a**) GCD curves for both samples at 0.1 A g^−1^. (**b**) GCD curves of PoPD-MO. (**c**) Comparison of rate performance. (**d**) CV curves for both samples at 0.2 mV s^−1^. (**e**) CV curves of PoPD-MO at 1.0 mV s^−1^ for the fourth to sixth cycles. (**f**) Cycling performance at 3 A g^−1^. (**g**) Ragone diagram for PoPD-MO.

The electrochemical properties of MO and PoPD-MO were tested using MO and PoPD-MO as cathodes. Figure 3a depicts the constant current charging–discharging curves (GCD) of MO and PoPD-MO at 0.1 A g^−1^, in which PoPD-MO has a longer and more stable discharge plateau, which suggests that the PoPD is embedded into the interlayer of the MO as a support within the interlayer, which improves the stabilization of the cathode, ultimately leading to better performance of PoPD-MO than MO. In addition, Figure 3b and Figure S6 (Supplementary Materials) depict the GCD curves of PoPD-MO and MO at different current densities. As the current density rises, the polarization phenomenon of MO is faster. In contrast, the polarization of PoPD-MO is weaker, which proves that the material has good reversibility and excellent storage performance. Figure 3c shows the multiplicity curves for both cathodes. As the current density rises from 0.1 to 3.0 A g^−1^, PoPD-MO discharge capacities are recorded at 359, 341, 296, 229, 180, 139, and 93 mAh g^−1^, yet the discharge capacities of MO are 244, 205, 181, 154, 127, 108, and 67 mAh g^−1^. When the current density was reduced to 0.1 A g^−1^, the capacity of PoPD-MO was still stabilized at 373 mAh g^−1^, which was better than that of MO (237 mAh g^−1^), indicating that PoPD played a supportive role in the intercalation layer and slowed down the structural collapse. Meanwhile, we also tested the XRD of the anode of the PoPD-MO//Zn battery after 2100 long cycles, as shown in Figure S7 (Supplementary Materials), and the main lattice of the PoPD-MO material did not change significantly during the phase evolution after long cycling. These results indicate that the PoPD-MO//Zn cell maintains excellent multiplicity performance, which meets the requirements of both high capacity and fast charging and discharging. Cyclic voltammetry (CV) analyses were conducted at 0.2 mV s^−1^ for two samples, and the curves obtained are shown in Figure 3d. It can be observed that both cathodes exhibit similar redox peaks, and the reduction peaks at 1.21 and 1.36 V align with Zn^2+^ and H^+^ insertion/extraction and Mn^4+^ reduction to Mn^3+^ [47]. Compared with pure MO, PoPD-MO has a larger cyclic volt-ampere area, suggesting that it also has a larger electrochemical capacity. As the scanning rate rises to 1 mV s^−1^, the CV curves of PoPD-MO begin to overlap progressively from the fourth to the sixth cycle (Figure 3e). It indicates that the PoPD-MO electrode material is highly reversible and stable, which can accelerate the repeated embedding and detaching of Zn^2+^ in the nanostructures. In contrast, the overlap of the CV curves of MO is slightly worse (Figure S8, Supplementary Materials). To assess the cycling stability and capacity retention of the materials, both underwent a series of cycling tests. Illustrated in Figure 3f is the cycling performance at a current density of 3 A g^−1^. Unlike the MO material, the PoPD-MO shows a significantly slower rate of capacity decline. Remarkably, after an exhaustive 2100 cycles, PoPD-MO still retains a discharge-specific capacity of 84.9 mAh g^−1^, a respectable 91.3% of its initial capacity. What is more, the Coulombic efficiency approaches 100%. Pure MO, however, ends its discharge much earlier, with a sharp drop in capacity after only 1400 cycles, and the retention rate is only 82.2% of the initial value. For PoPD-MO, the embedding of PoPD enhances the stabilization of the structure, accelerates the reaction kinetics, and offers more electrochemically active sites, which ultimately leads to an increase in capacity. Figure S9 (Supplementary Materials) shows the cycling performance of both MO and PoPD-MO at a current rate of 0.1 A g^−1^. Notably, the initial specific capacity of the PoPD-MO cathode was 275 mAh g^−1^, which exceeds the same conditions of MO. After 85 cycles, the capacity of MO plummeted, while the capacity of PoPD-MO still maintained an increasing trend. Even after 230 cycles, the capacity retention rate was still at 80%. Therefore, PoPD-MO exhibits better electrochemical performance than MO in ZIBs. Figure 3g showcases the Ragone plot, illustrating the power density of PoPD-MO alongside its energy density. PoPD-MO demonstrates an impressive energy density of 394.7 Wh/kg at a power density of 106.6 W/kg. Even when pushed to higher power densities, such as 788.9 W/kg, it maintains a robust energy density of 118.3 Wh/kg. These figures outshine the majority of vanadium- and manganese-based materials documented in recent studies, as outlined in Table S1 (Supplementary Materials). Overall, PoPD-MO exhibits excellent overall performance among other manganese-based materials that have been reported (Table S2, Supplementary Materials).

**Figure 4:**
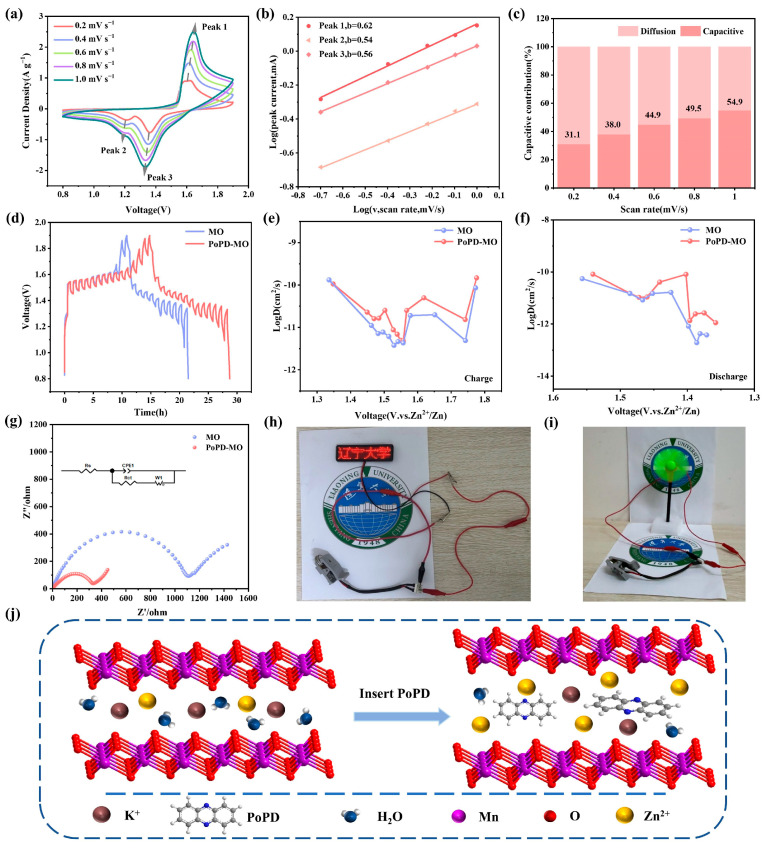
(**a**) CV curves of PoPD-MO between 0.2 and 1.0 mV s^−1^. (**b**) Relationship between log(*i*) and log(*v*) corresponding to the redox peaks of the PoPD-MO electrode. (**c**) Capacitance contribution at varying scanning rates. (**d**–**f**) GITT curves and corresponding Zn^2+^ diffusion coefficients of PoPD-MO and MO. (**g**) Nyquist plots of the PoPD-MO and MO cathodes before cycling. (**h**,**i**) Images of a battery powering both an LED and a fan. (**j**) Schematic of Zn^2+^ diffusion after PoPD intercalation.

As depicted in Figure 4a and Figure S10 (Supplementary Materials), the CV curves of both cathodes were examined across scan rates between 0.2 and 1.0 mV s^−1^. With heightened scan rates, the oxidation peaks shift towards increased potentials, while the reduction peaks migrate to lower potentials. A set of *b* values is calculated by correlating the scan rate (*v*) with the current (*i*). The relationship between them can be expressed as follows [48]:

(1) $$i\;=av^{b}$$

or

(2) log(i)= blog(v)+log(a)

Using log(*v*) and log(*i*) as the horizontal and vertical coordinates, the fitted slope is the *b* value, and if *b* = 0.5, it means that the electrochemical procedure is dominated by diffusion control, and if *b* = 1, it means that the process is predominantly capacitively led in its behavior [49,50]. Figure 4b shows that the *b* values corresponding to Peak 1, Peak 2, and Peak 3 of PoPD-MO are 0.62, 0.54, and 0.56. Meanwhile, Figure S11 (Supplementary Materials) shows that the *b* values for Peaks 1, 2, and 3 of MO stand at 0.508, 0.559, and 0.554, indicating that this reaction process is mainly controlled by the diffusion process. In addition, the contribution of the two controls can be calculated according to the equation *i* = *k*_1_*v* + *k*_2_*v*^1/2^ [51], where *k*_1_ is the capacitance contribution coefficient and *k*_2_ is the diffusion coefficient, Figure 4c and Figure S12 (Supplementary Materials). 

The capacitance contribution of both cathode materials increases with the scan rate. Specifically, the capacitive contribution of PoPD-MO was calculated between 31.1% and 54.9% for scanning rates between 0.2 and 1.0 mV s^−1^, both of which are slightly lower than the capacitance contribution of MO (from 33.6% to 67.3%). It can be seen that the capacitive contributions of both MO and PoPD-MO are relatively low, indicating that the diffusion works in conjunction with the capacitance control behavior [38]. The introduction of PoPD increases the electrochemical active sites, expands the space for Zn^2+^ movement, and enhances ion transport, which ultimately realizes the excellent multiplicity performance and high reversible capacity of ZIBs.

To elucidate the differences in Zn^2+^ diffusion kinetics between PoPD-MO and MO and to demonstrate their impact on energy storage performance, we conducted galvanostatic intermittent titration technique (GITT) tests (Figure 4d–f). The calculated average D_Zn_^2+^ values for the PoPD-MO battery during charging and discharging were 3.459 × 10^−11^ and 2.470 × 10^−11^ cm^2^ s^−1^, respectively, which are higher than those of the MO battery (2.552 × 10^−11^ and 1.235 × 10^−11^ cm^2^ s^−1^). Therefore, PoPD-MO exhibits a larger diffusion coefficient, facilitating the rapid diffusion of Zn^2+^ and resulting in superior electrochemical performance. Figure 4g illustrates the electrochemical impedance spectroscopy (EIS) curves of the two materials and their equivalent circuit diagrams. It can be seen that the electrochemical impedance spectral curves of both PoPD-MO and MO consist of a semicircle and a diagonal line. Where the semicircle is attributed to the charge transfer resistance (R_ct_) and the slash is attributed to the diffusion of Zn^2+^ to the electrode. The calculated charge transfer resistances of the MO cathode and PoPD-MO cathode are 1080 Ω and 314 Ω, respectively, which indicates that the insertion of PoPD greatly reduces the charge transfer resistance and enhances the conductivity of the cathode material. The decrease in charge transfer resistance may be attributed to the introduction of PoPD, which enlarges the interlayer spacing of the MO and thus improves the ion transfer efficiency. In addition, Figure S13 (Supplementary Materials) shows the Nyquist plots before and after PoPD-MO cycling, and by fitting and calculating the EIS curves, we found that the R_ct_ value changed from 314 Ω to 336 Ω after 100 cycles of PoPD-MO, which indicated that there was no significant change in the charge transfer resistance of the cathode of PoPD-MO after cycling. When using this material as the positive electrode to assemble a battery for testing, the results show that two button batteries connected in series can continuously light up an LED signboard (Figure 4h) and one button battery can make a small fan rotate continuously (Figure 4i), indicating that the PoPD-MO//Zn battery has a stable voltage, enabling it to supply power stably and showing great potential to become an excellent energy storage element. Thus, it can be seen that the PoPD-MO cathode performs very well in ZIBs. Combined with Figure 4j, we can infer that PoPD incorporation expands the interlayer distance of the cathode material, which not only accelerates the diffusion of Zn^2+^ and H^+^, but also enhances the stability of the material, and ultimately realizes the improvement in the battery capacity.

## 3. Conclusions

In conclusion, this study presents and prepares, for the first time, electrochemically active poly(o-phenylenediamine)-intercalated MnO_2_ composites which are modified by inserting the organic polymer PoPD into MO. On the one hand, PoPD plays a supporting role between the MO layers, enlarges the layer spacing, and enhances the electrical conductivity, providing space for the embedding of more Zn^2+^. On the other hand, the C=N bond in the PoPD coordinates with the divalent Zn ions, which can realize the Zn storage while maintaining the stability of the voltage platform. As a result, the prepared PoPD-MO cathode has excellent multiplication capability and superb cycling stability. Electrochemical tests show that the PoPD-MO//Zn cell can provide a capacity of 359 mAh g^−1^ at 0.1 A g^−1^, and maintain 91.3% of its capacity even after 2100 cycles at a high current density of 3 A g^−1^. Therefore, this work not only reveals the excellent electrochemical capability of PoPD-MO cathodes, but also provides a new method for the modification of other electrode materials.

## Data Availability

The original contributions presented in the study are included in the article/supplementary material, further inquiries can be directed to the corresponding author.

## Supplementary Materials

The following supporting information can be downloaded at: https://www.mdpi.com/article/10.3390/polym17081003/s1, Figure S1: High-resolution XPS analysis of MO (a) C 1s, (b) Mn 2p, (c) O 1s spectra; Figure S2: The SEM image of MO; Figure S3: EDS mappings of MO; Figure S4: The EDS image of the PoPD-MO cathode; Figure S5: TEM and HR-TEM images of MO; Figure S6: GCD curves of the MO cathode at different current densities from 0.1 to 3.0 A g^−1^; Figure S7: XRD image after 2100 cycles of PoPD-MO; Figure S8: CV curves of the MO at 1.0 mV s^−1^; Figure S9: Cycling performance of MO and PoPD-MO at 0.1 A g^−1^; Figure S10: CV curves at different scan rates; Figure S11: The corresponding dependence of log(*i*) versus log(*v*) for the three peaks of MO; Figure S12: Contribution ratios of capacitive control behavior and diffusion control behavior at 0.2–1.0 mV s^−1^; Figure S13: Nyquist plots of PoPD-MO cathode before and after 100 cycles; Table S1: Comparison table of energy density of zinc ion energy storage devices in different systems [19,52,53,54,55,56]; Table S2: The comparison of electrochemical properties of PoPD-MO with the previously reported cathode materials for zinc-ion batteries [34,39,57,58,59,60].
