# Supplementary material for: Active Poly(o-phenylenediamine)-Intercalated Layered δ-MnO2 Cathode for High-Performance Aqueous Zinc-Ion Batteries
Source: Polymers (Basel). 2025 Apr 8;17(8):1003. doi: 10.3390/polym17081003 (PMC12030697; doi:10.3390/polym17081003)
Supplement: Supplementary file 1 [file polymers-17-01003-s001.zip › polymers-3543478-supplementary.pdf]

Supplementary Materials

Active Poly(o-phenylenediamine)-Intercalated Layered δ-MnO2 Cathode for High-Performance Aqueous Zinc-Ion Batteries

Ziqian Yuan, Bosi Yin, Wenhui Mi, Minghui Liu and Siwen Zhang *

Institute of Clean Energy Chemistry, Key Laboratory for Green Synthesis and Preparative Chemistry of Advanced Materials, College of Chemistry, Liaoning University, Shenyang 110036, China

*** Correspondence:** zhangsiwen@lnu.edu.cn (S.-W. Zhang)

Materials and Methods

1. Synthesis of PoPD/MO and MO

KMnO4 (4 mmol) and $C_6H_8N_2$ (0.3 mmol) were dissolved in 30 mL of deionized water. The mixture was stirred for 1 h. Then, the mixed solution was adjusted to pH = 4 with HCl and stirred for another 30 min. The prepared solution was transferred to a polytetrafluoroethylene-lined autoclave and carried out at 200 °C for 24 h. The solution cooled to room temperature was washed three times with deionized water and anhydrous ethanol by centrifugation, and then vacuum dried overnight. The resulting powder sample was denoted as PoPD/MO. Pure MO was prepared without o-phenylenediamine under the same preparation conditions.

2. Manufacture of CR2032 coin cell batteries

The working electrode was prepared by dispersing MO or PoPD/MO, Super P and polyvinylidene fluoride (PVDF) in N methyl pyrrolidone (NMP) in the ratio of 8:1:1. Then, the mixed slurry was applied to a piece of carbon paper with a spatula, and then dried in a vacuum oven at 60 °C for 12 h. The dried working electrode and anode were cut into 12 mm discs, and the cathode was loaded with active materials in the range of 1.0 to 1.5 mg. The CR2032 coin cell was made by using the prepared working electrode as the cathode, the zinc metal as the anode, and the glass fibers as the diaphragm, with 2 M $Zn(CF_3SO_3)_2$ + 0.1 M $MnSO_4$ as electrolyte.

3. Electrochemical performance testing

The electrochemical performance of zinc ion batteries was tested at room temperature by the NEWARE Battery Test System, a multichannel electrochemical

analyzer (VMP3, Bio-Logic-Science Instruments), and a CHI (760E, Chenhua), which includes a constant-current charge/discharge (GCD), galvanostatic intermittent titration technique (GITT), cyclic voltammetry (CV), and electrochemical impedance spectroscopy (EIS) electrochemical workstations.

4. Material characterization

XRD tests were performed on both samples using Rigaku Ultima IV and Cu Kα radiation with scans in the angular range of $10° \leq 2\theta \leq 80°$. The chemical composition of the samples was characterized by XPS (Thermo Scientific K-Alpha). SEM images and TEM images were observed using a scanning electron microscope (ZEISS Gemi-niSEM 300) and a transmission electron microscope (JEM-2100). Raman spectros-copy (Horiba LabRAM HR Evolution) was utilized to identify the chemical structure of the samples.

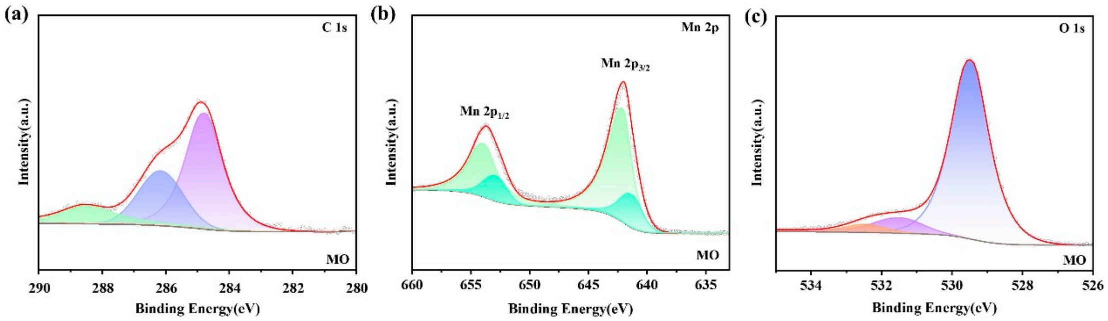

Figure S1. High-resolution XPS analysis of MO (a) C 1s, (b) Mn 2p, (c) O 1s spectra.

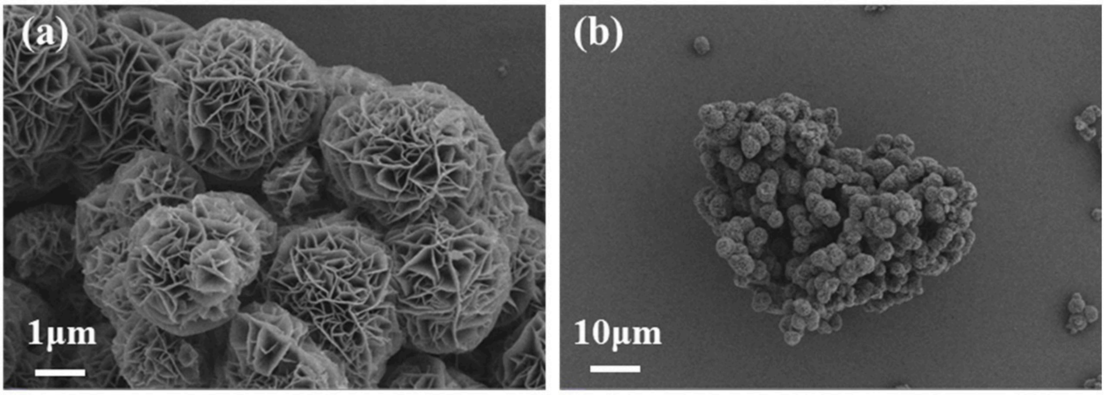

Figure S2. The SEM image of MO.

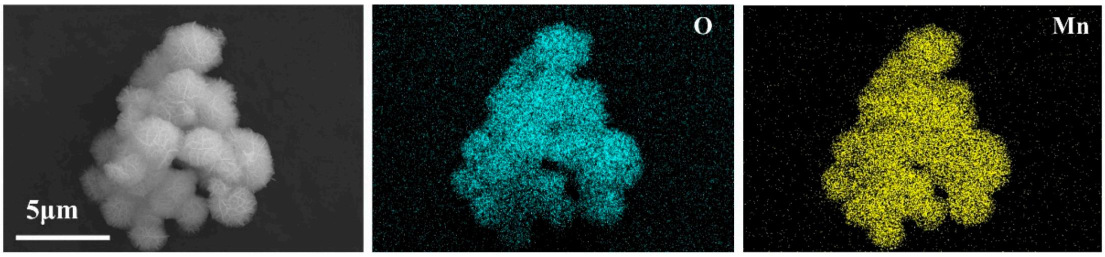

Figure S3. EDS mappings of MO.

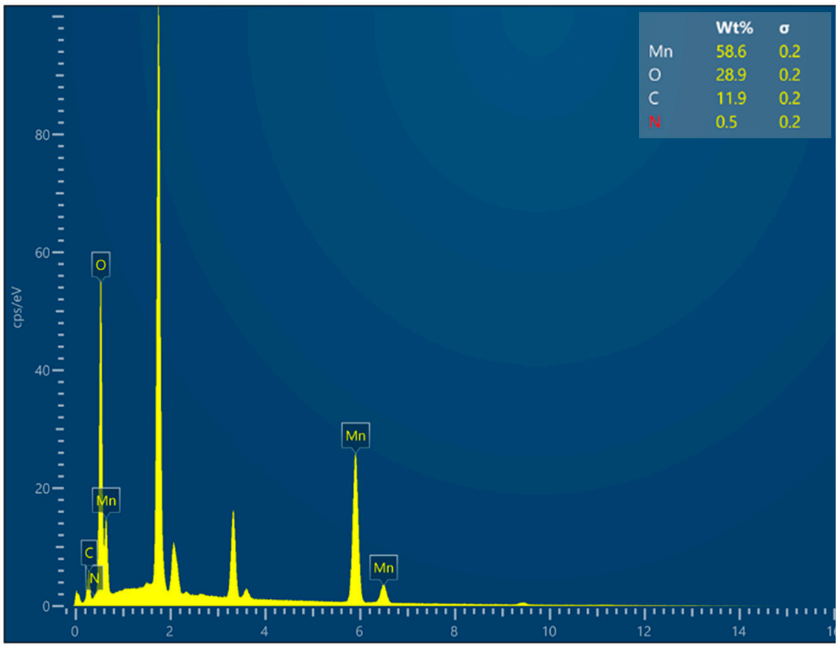

Figure S4. The EDS image of the PoPD-MO cathode.

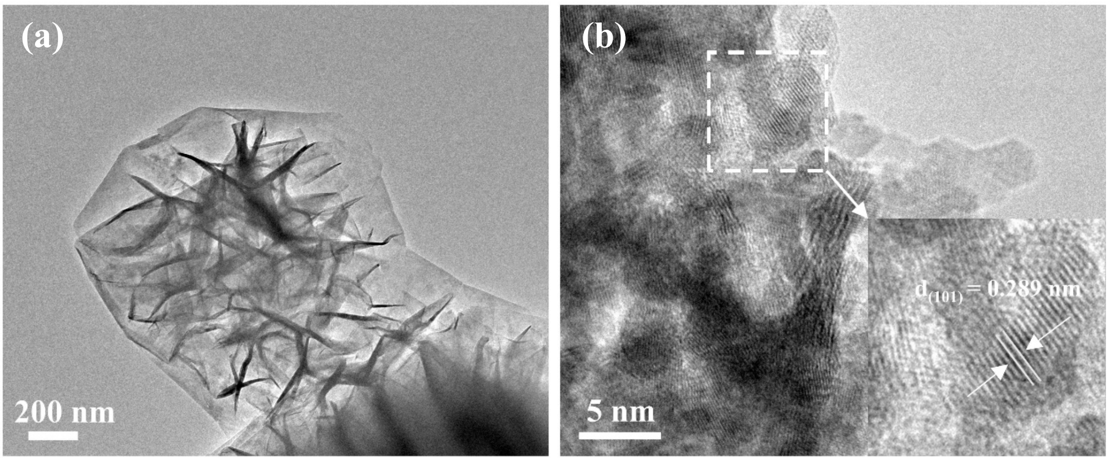

Figure S5. TEM and HR-TEM images of MO.

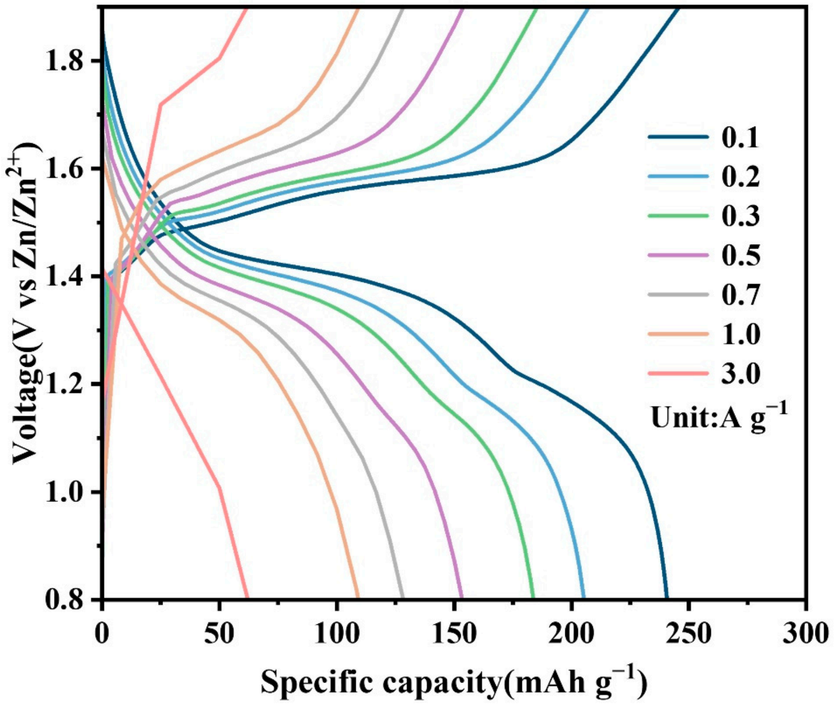

Figure S6. GCD curves of the MO cathode at different current densities from 0.1 to 3.0 A g^{-1}.

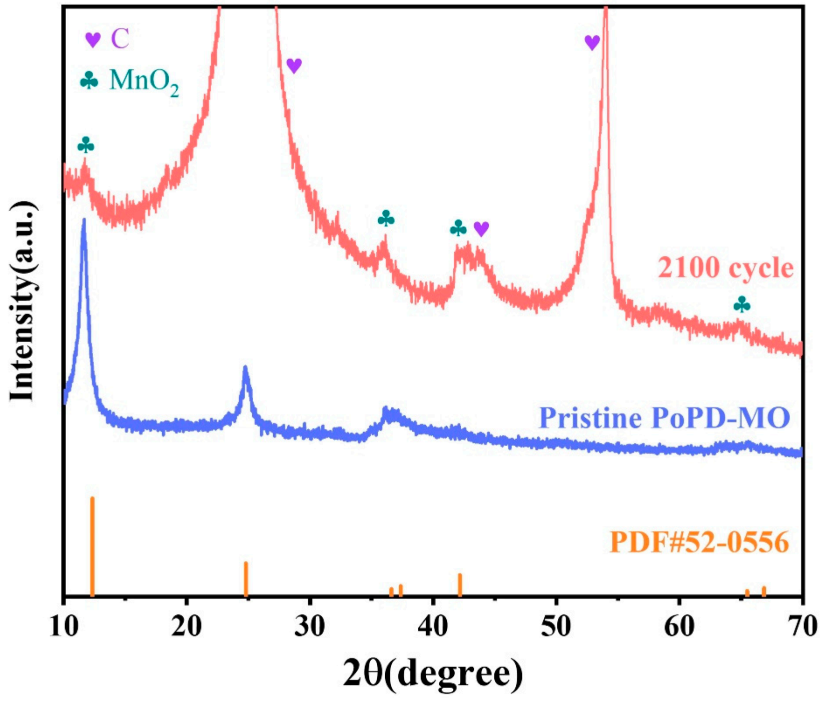

Figure S7. XRD image after 2100 cycles of PoPD-MO.

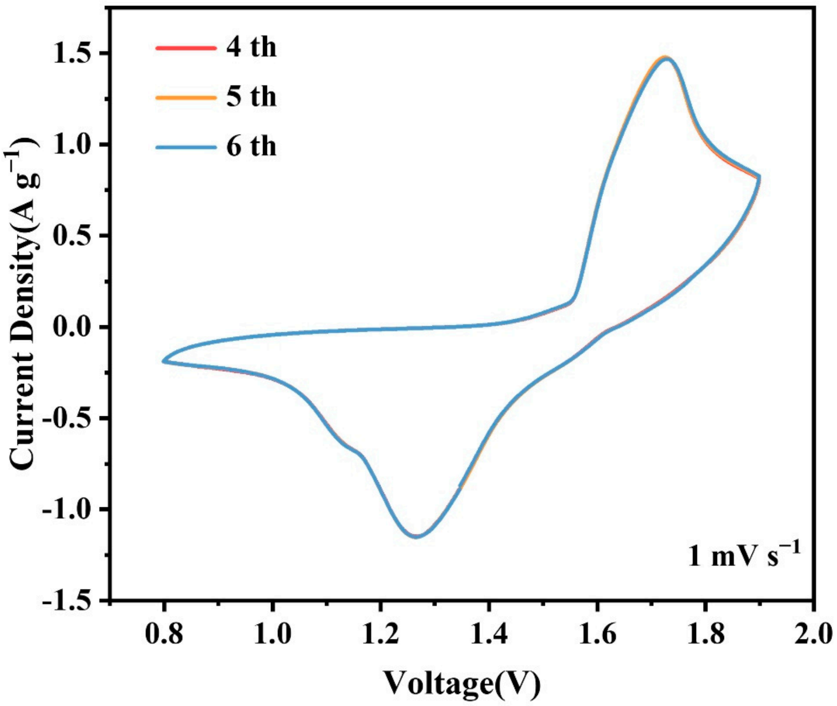

Figure S8. CV curves of the MO at 1.0 mV s^{-1}.

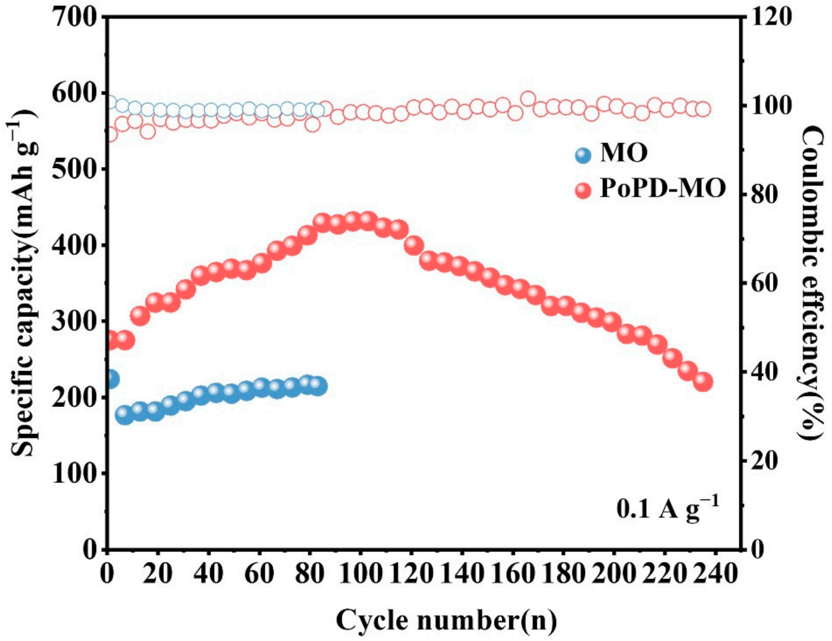

Figure S9. Cycling performance of MO and PoPD-MO at 0.1 A g^{-1}.

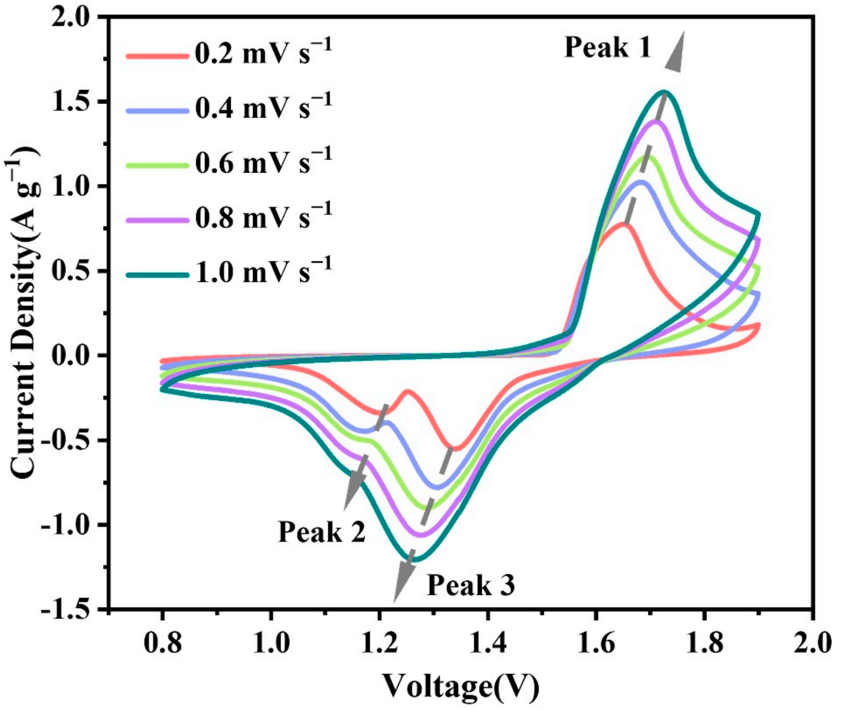

Figure S10. CV curves at different scan rates.

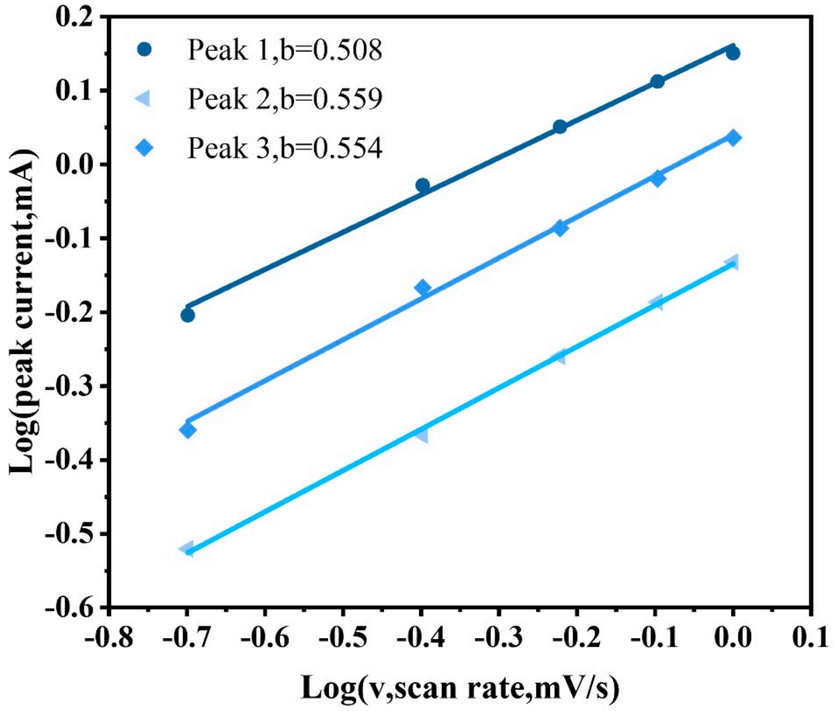

Figure S11. The corresponding dependence of log(i) versus log(v) for the three peaks of MO.

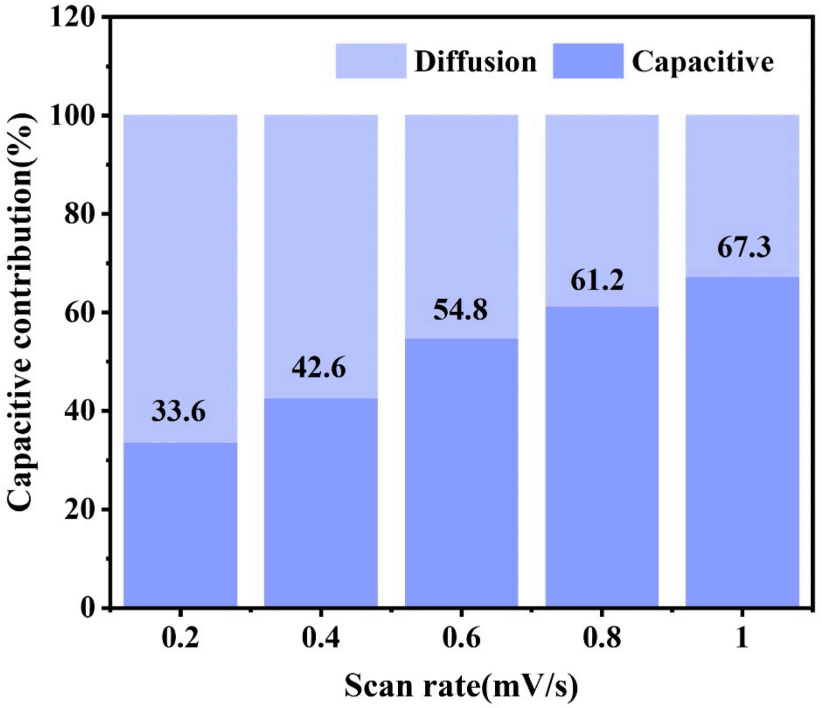

Figure S12. Contribution ratios of capacitive control behavior and diffusion control behavior at 0.2−1.0 mV s^{-1}.

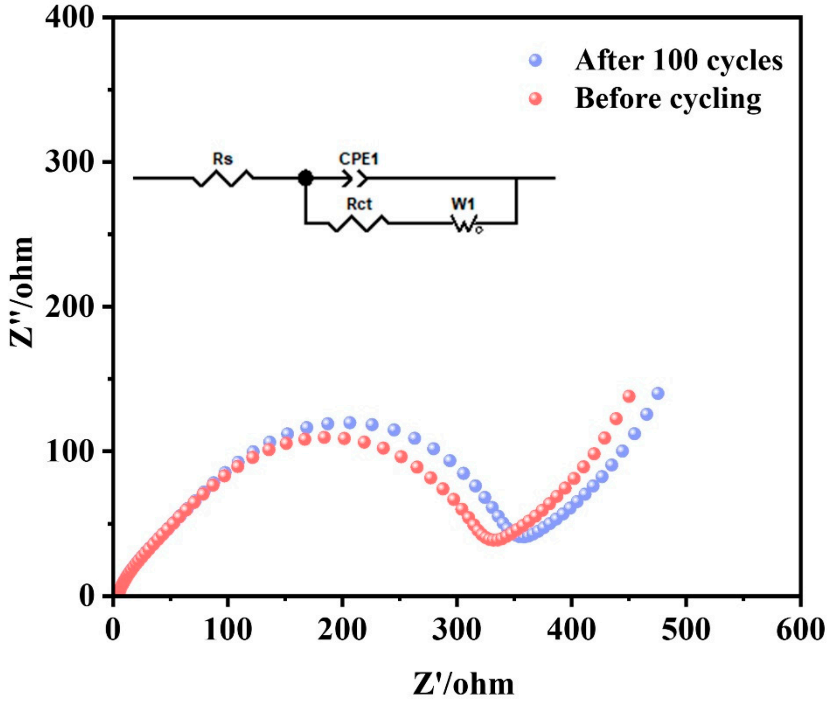

Figure S13. Nyquist plots of PoPD-MO cathode before and after 100 cycles.

Table S1. Comparison table of energy density of zinc ion energy storage devices in different systems.

Cathode//Anode	Electrolyte	Voltage (V)	Energy density (Wh/kg)	Ref.
PoPD-MnO_2//Zn	2 M $Zn(CF_3SO_3)_2$ +0.1 M $MnSO_4$	0.8~1.9	394.7	This work
$ZnMn_2O_4$//Zn	3 M $Zn(CF_3SO_3)_2$	0.8~1.9	170	[1]
CS@MnO_2−15//Zn	2 M $ZnSO_4$+0.1 M $MnSO_4$	0.8~1.8	280	[2]
β-MnO_2//Zn	3 M $Zn(CF_3SO_3)_2$ +0.1 M	0.8~1.9	254	[3]
δ-MnO_2//Zn	1 M $ZnSO_4$	1.0~1.8	185	[4]
$Zn_{0.25}V_2O_5\cdot nH_2O$//Zn	1 M $ZnSO_4$	0.5~1.4	170	[5]
$H_2V_3O_8$ Nanowire/ Graphene//Zn	3 M $Zn(CF_3SO_3)_2$	0.2~1.6	168	[6]

Table S2. The comparison of electrochemical properties of PoPD-MO with the previously reported cathode materials for zinc-ion batteries.

Cathode materials	Voltage range (V)	Specific capacitance	Rate capacity retention	Ref.
PoPD-MnO_2	0.8~1.9	359 $mAh\cdot g^{-1}$ @0.1 $A\cdot g^{-1}$	229 $mAh\cdot g^{-1}$ @0.5 $A\cdot g^{-1}$ 139 $mAh\cdot g^{-1}$ @1 $A\cdot g^{-1}$	This work
a-AMO	0.7~1.9	290.2 $mAh\cdot g^{-1}$ @0.1 $A\cdot g^{-1}$	104.2 $mAh\cdot g^{-1}$ @1.0 $A\cdot g^{-1}$	[7]
MnO_2-Bet	0.8~1.8	306 $mAh\cdot g^{-1}$ @0.1 $A\cdot g^{-1}$	306 $mAh\cdot g^{-1}$ @0.1 $A\cdot g^{-1}$	[8]
GC-dMO	0.8~1.9	267 $mAh\cdot g^{-1}$ @0.05 $A\cdot g^{-1}$	60 $mAh\cdot g^{-1}$ @5.0 $A\cdot g^{-1}$	[9]
Al-MnO_2	0.8~1.8	210 $mAh\cdot g^{-1}$ @0.1 $A\cdot g^{-1}$	44 $mAh\cdot g^{-1}$ @2.0 $A\cdot g^{-1}$	[10]
PEDOT-MnO_2	1.0~1.8	300 $mAh\cdot g^{-1}$ @0.2 $A\cdot g^{-1}$	122 $mAh\cdot g^{-1}$ @2.0 $A\cdot g^{-1}$	[11]
Mo-MnO_2	0.9~1.8	327 $mAh\cdot g^{-1}$ @0.2 $A\cdot g^{-1}$	107 $mAh\cdot g^{-1}$ @1.0 $A\cdot g^{-1}$	[12]

Reference

[1] Zhang, N.; Cheng, F.; Liu, Y.; Zhao, Q.; Lei, K.; Chen, C.; Liu, X.; Chen, J. Cation-Deficient Spinel $ZnMn_2O_4$ Cathode in $Zn(CF_3SO_3)_2$ Electrolyte for Rechargeable Aqueous Zn-Ion Battery. *J.Am.Chem.Soc.* **2016**, *13839*, 12894−12901.

[2] Yu, B.; Lu, L.; He, Y.; Dai, X.; Wang, Y.; Wang, T.; Chong, S.; Liu, L.; Liu, Y.; Tan, Q. Hierarchical Porous CS@Ce-MnO_2 as Cathode for Energy-Dense and Long-Cycling Flexible Aqueous Zinc-Ion Batteries. *J. Colloid Interface Sci.* **2024**, *654*, 56-65.

[3] Zhang, N.; Cheng, F.; Liu, J.; Wang, L.; Long, X.; Liu, X.; Li, F.; Chen, J. Rechargeable Aqueous Zinc-Manganese Dioxide Batteries with High Energy and Power Densities. *Nat. Commun.* **2017**, *81*, 405.

[4] Kamenskii, M. A.; Volkov, F. S.; Eliseeva, S. N.; Holze, R.; Kondratiev, V. V. Comparative Study of PEDOT- and PEDOT:PSS Modified δ-MnO_2 Cathodes for Aqueous Zinc Batteries with Enhanced Properties. *JES* **2023**, *1701*, 010505.

[5] Kundu, D.; Adams, B. D.; Duffort, V.; Vajargah, S. H.; Nazar, L. F. A High-Capacity and Long-Life Aqueous Rechargeable Zinc Battery Using a Metal Oxide Intercalation Cathode. *Nat. Energy* **2016**, *110*, 16119.

[6] Pang, Q.; Sun, C.; Yu, Y.; Zhao, K.; Zhang, Z.; Voyles, P. M.; Chen, G.; Wei, Y.; Wang, X. $H_2V_3O_8$ Nanowire/Graphene Electrodes for Aqueous Rechargeable Zinc Ion Batteries with High Rate Capability and Large Capacity. *Adv. Energy Mater.* **2018**, *819*, 1800144.

[7] Yang, L.; Zhang, J.; Zhou, X.; Liu, Y. Amorphous Aluminum-Doped Manganese Oxide Cathode with Strengthened Performance for Aqueous Zinc-Ion Batteries. *J ALLOY COMPD* **2025**, *1012*, 178502.

[8] Zhang, A.; Yin, X.; Saadoune, I.; Wei, Y.; Wang, Y. Zwitterion Intercalated Manganese Dioxide Nanosheets as High-Performance Cathode Materials for Aqueous Zinc Ion Batteries. *Small.* **2024**, *2042*, 2402811.

[9] Naresh, N.; Eom, S.; Lee, S. J.; Jeong, S. H.; Jung, J.-W.; Jung, Y. H.; Kim, J.-H. Disordered Structure and Reversible Phase Transformation from K-Birnessite to Zn-Buserite Enable High-Performance Aqueous Zinc-Ion Batteries. *EEM* **2024**, *73*, e12640.

[10] Chomkhuntod, P.; Sawangphruk, M. Understanding the Effect of Pre-Intercalated Cations on Zn-Ion Storage Mechanism of Layered Birnessite Manganese Oxide for Aqueous Zn-ion Batteries. *ECS Meeting Abstracts.* **2022**, *MA2022-011*, 25.

[11] Wang, L.; Wang, X.; Song, B.; Wang, Z.; Zhang, L.; Lu, Q. Facile in Situ Synthesis of PEDOT Conductor Interface at the Surface of MnO_2 Cathodes for Enhanced Aqueous Zinc-Ion Batteries. *SURF INTERFACES* **2022**, *33*, 102222.

[12] Wang, Z.; Han, K.; Wan, Q.; Fang, Y.; Qu, X.; Li, P. Mo-Pre-Intercalated MnO_2 Cathode with Highly Stable Layered Structure and Expanded Interlayer Spacing for Aqueous Zn-Ion Batteries. *ACS Appl. Mater.* **2023**, *151*, 859-869.
